# Physiotherapy for Piriformis Syndrome Using Sciatic Nerve Mobilization and Piriformis Release

**DOI:** 10.7759/cureus.32952

**Published:** 2022-12-26

**Authors:** Sidra Ahmad Siraj, Ragini Dadgal

**Affiliations:** 1 Physiotherapy, Ravi Nair Physiotherapy College, Datta Meghe Institute of Higher Education and Research, Wardha, IND; 2 Neurophysiotherapy, Ravi Nair Physiotherapy College, Datta Meghe Institute of Higher Education and Research, Wardha, IND

**Keywords:** visual analog scale, physical therapy, piriformis release, sciatic nerve mobilization, numerical pain rating scale

## Abstract

Piriformis syndrome is also synonymous with sciatica or buttock pain. This is a condition where the muscle irritates the sciatic nerve. This nerve passes above, below, or in between the piriformis muscle piercing it. The muscle tightens or shortens, thus compressing the nerve and disturbing the impulses passing from it. The sciatic nerve is a combination of nerve roots from L4 to S3. Piriformis works as a lateral rotator and is a synergistic muscle of the flexor and abductor group. Females most commonly present with piriformis syndrome than males. Many causative factors are responsible for the compression or impingement of the sciatic nerve, one of which is piriformis syndrome. Tingling, numbness, and pain are most often felt by patients when they have compression of any of the nerves. Many physiotherapy techniques have been found to be effective in managing this problem. Techniques like nerve mobilization, stretching, myofascial release, deep friction massage, and many more have been studied by authors describing their effects in the treatment of piriformis syndrome. Neural mobilization consists of two techniques, nerve gliding and nerve tensioning. Studies have found that the gliding technique produces less strain on the nerve than the tensioning technique. Piriformis stretch reduces the tightening, which has caused the impingement. Two techniques have been used for this stretch, stretching with hip flexion over 90 degrees and hip flexion under 90 degrees. This review focuses on the different advances in treating piriformis syndrome.

## Introduction and background

The nerve is a structure that consists of bundles of axons arranged in such a manner that they connect the central nervous system and peripheral nervous system. Its outermost layer, called the epineurium, contains a group of fascicles that are covered by the perineurium. The fascicle is made up of bunches of nerve fibers, which are either myelinated or nonmyelinated [1]. In the peripheral nerve, Schwann cells are responsible for myelin formation and oligodendrocytes do the same in the central nervous system. They wrap their cell membranes around the axons to form a multi-layered membranous sheath that provides insulation and support to the axons [2]. They are responsible for transmitting electrical impulses along the length of the nerve; if myelin is damaged, these impulses slow down [3].

The sciatic nerve is a peripheral nerve that combines five spinal nerves L4, L5, S1, S2, and S3 [4]. It is a mixed type of nerve having both motor and sensory fibers. These merge deep in the gluteal region to form a thick long sciatic nerve. At the buttock, it can pass either above, below, or between the piriformis muscle piercing it, and then traverse through the posterior of the thigh. Prior to entering the popliteal fossa, it divides into the common peroneal nerve, which continues through the lateral of the leg, and the tibial nerve, which journeys through the posterior of the leg [5]. The piriformis muscle begins from the inferior surface of the sacrum and ends on the superior border of the greater trochanter of the femur [6]. This muscle acts as a lateral rotator, weak flexor, and feeble abductor of the hip joint, and it also provides steadiness during standing and ambulation. It acts as a lateral rotator when the hip joint is flexed to less than or equal to 60ᵒ. Once the angle of hip flexion goes beyond 60ᵒ, the function of the piriformis muscle changes; that is, it becomes an internal rotator. Thus, the piriformis stays active during any sort of sitting position, whether high or cross-sitting, though its role changes in different movements [3].

There are two types of classification for peripheral nerve degeneration, which are Sunderland’s classification and Seddon’s classification [7]. There are three types of nerve injury in Seddon’s classification. The first type is neuropraxia: a temporary physiological block produced by ischemia due to compression with no Wallerian degeneration and recovery in minutes to days. The second type is axonotmesis; the interior architecture of the nerve is well-preserved, but axons are damaged, and Wallerian degeneration occurs; recovery is in months. Axon redevelops at a rate of 1mm/day. The final type is neurotmesis, where the nerve structure is disrupted as a result of cutting, severe scarring, or chronic severe compression; there is complete sensory and motor and sympathetic loss, which recovers in months only after surgery [8]. Sunderland's classification has five degrees: 1ᵒ same as neuropraxia, 2ᵒ and 3ᵒ same as axonotmesis, and 3ᵒ, 4ᵒ, and 5ᵒ same as neurotmesis [9,10].

If the piriformis muscle spasms, it causes compression of the sciatic nerve leading to pain, tingling, and numbness in its course, which are almost similar to symptoms of sciatica [11,12]. This usually occurs in piriformis syndrome. Symptoms include tingling, numbness, and pain which extends from the back of the foot. Pain is intermittent in nature and aggravates on movements like jumping, running, etc [13]. Piriformis syndrome is also synonymous with sciatica or buttock pain [14,15]. Some of the manual tests performed for the diagnosis of piriformis syndrome are the flexion, adduction, and internal rotation (FAIR) test, Pace maneuver, and Beatty’s test [16].

Treatment for piriformis syndrome

Conservative treatment that provides symptomatic relief includes muscle relaxants, nonsteroidal anti-inflammatory drugs, or drugs that relieve neuropathic pain [17]. Keeping the muscle in a relaxed position reduces the tension placed on the nerve, thereby alleviating pain. As the muscle contracts, the nerve is compressed, so relaxation of muscle that is keeping it in a position where it does not act releases the tension on nerve. One of the methods for relaxing this muscle is piriformis stretch. Standing or sitting positions can be used for stretching by placing the hip in adduction, internal rotation, and flexion with the knee in flexion too, as done in the FAIR test in which the subject lies on the side with the affected leg facing the therapist and the therapist flexes the hip and knee till 90 degrees and internally rotates the hip joint therefor placing stretch on the piriformis and compressing the sciatic nerve resulting in pain at the buttocks region. Prior to stretching, application of heat helps in further relaxing the muscle [18]. Releasing trigger points via myofascial release in the buttock region reduces spasm of the piriformis muscle and relieves pain [19]. Deep friction massage significantly decreases pain and discomfort felt while performing daily activities. Deep tissue mobilization with regular stretching sessions decreases the compression of sciatic nerve with proper posture and movement awareness so as to prevent unwanted spasm in the muscle. Regular stretching with awareness of movement decreases worsening of the condition [20]. Cold and heat application on the irritated and tight piriformis muscle before and after physical therapy helps in lessening the discomfort felt after therapy [21]. Botulinum toxin injected in piriformis muscle along with physiotherapy maneuver decreases the irritation on the sciatic nerve and radiating pain along the nerve by relaxing the tensed muscle. These steroids help in recovery of the nerve from compression [22].

Neural mobilization or nerve mobilization is a technique used for treating nerves that are irritated, inflamed, or adherent. It consists of two methods: nerve gliding and nerve tensioning [23]. Neural mobilization helps in relieving the nerve and restoring the normal flow of impulse through it, thereby reducing the symptoms [24]. Nerve gliding involves using two joints, and movement is performed in such a way that one joint is moved causing elongation of neural structures at one end and shortening at the other end of the joint simultaneously [25]. Nerve tensioning involves executing joint motions that lengthen the nerve till symptoms develop and then mobilizing with the articular joint distal to where symptoms are thought to originate. Scholars say that the gliding technique provides less strain to the nerve than the tensioning technique, whereas nerve excursion is more in the gliding technique than in the tensioning technique without the potentially large increases in nerve strain [26].

As the piriformis muscle spasms, the symptoms of sciatica are most commonly observed. This can also be treated by piriformis stretching. Stretching enhances flexibility, physical performance, injury prevention, and muscular discomfort [27]. Few of the many types of piriformis stretching are SFCO stretching with coxal articulation flexion over 90° and muscle energy technique (MET) application (SFCU) stretching with coxal articulation flexion under 90°. The patient rests supine with both legs bent and one leg placed on the contrary side knee that will be graded for the SFCO. The patient next bends his knee over 90° till he feels tightness in the same direction as the stretched leg's shoulder and holds this position for 30 seconds. For SFCU, the patient in the supine position crosses the leg to be stretched over to the opposite side knee. For 30 seconds, the individual must touch the exterior of the knee of the leg being stretched with the contrary side hand and press the knee toward the floor [28]. In order to perform MET, the subject lies in the supine position, bends the coxal articulation and knee joint of the leg being tested, and places the testing side foot outside the opposite side knee so that the foot contacts the ground. The coxal articulation's flexion angle should not exceed 60°.The therapist limits pelvic movement by placing one hand on the opposite side's anterior superior iliac spine. The therapist does abduction for 10 seconds with the opposite side hand outside the bent knee and applies a pushing pressure during the piriform muscle contraction. Following contraction, the therapist performs adduction until the resistance on the investigated leg is felt and is maintained for 20 seconds [29].

## Review

Methodology

This review paper was mainly compiled from PubMed, Scopus, Web of Science, Springer, and ScienceDirect.

A total of 50 articles were studied, and 35 were included in this article. These meta-analyses were chosen based on the following criteria: English language publication, human subjects, observational research, and comparative studies, including review articles. These articles were chosen for their content rather than their geographical location. The publications selected included mainly physiotherapy treatment for piriformis syndrome irrespective of age group or gender.

Discussion 

Kirschner et al. in their review studied the possible reasons resulting in piriformis syndrome and some of the physiotherapy managements involving stretching, followed by hip strengthening, lumbosacral stabilization, and myofascial release. They also found that injecting either a corticosteroid, local anesthetic, or botulinum toxin individually or in combination provided relief to those patients who did not respond to manual therapy. This has also been used for diagnostic purpose too [30].

A study on 250 patients by Michel et al. presenting with symptoms of piriformis syndrome was conducted with a number of examinations including the FAIR test, Freiberg maneuver, Beatty's test, and heel-contralateral knee maneuver. Deep transverse massage and pelvic-trochanter muscle stretch were combined with self-rehabilitation techniques and proprioceptive pelvic femoral exercises were designed for each individual. Treatment also included stretching with Onabotulinumtoxin A injections. Both the treatments showed a decrease in symptoms of piriformis syndrome [31].

Fusco et al. performed a case study on three patients suffering from piriformis muscle syndrome. Pain was evaluated on the numeric pain rating scale (NPRS), and treatment with ultrasound (US)-guided needle therapy was done. There was a significant decrease in pain in all three patients, and they concluded that US-guided needle therapy is a valid strategy treatment [32].

A comparative study on the effect of post-isometric relaxation (PIR) and reciprocal inhibition combined with conventional physiotherapy in three different groups of 15 patients each was performed by Bose et al. They found a reduction in pain, increased functional outcome, and improved hip range of motion. These results were much better in the group who received conventional therapy along with PIR provided they have decreased hip internal rotation with the knee at 0 degrees, pain on the visual analog scale (VAS): 3-6, only one die involvement of piriformis muscle, and at least three positive tests among the FAIR test, tonic external rotation of the hip, Freiberg test, Beatty's test, piriformis test, and Sign of Pace and Nagel [33].

Norbury et al. in their study assessed a 45-year-old female patient for piriformis syndrome. The pain on the VAS was 5/10, and the FAIR test was positive. A series of management was given, consisting of stretching, lifestyle modification, and corticosteroid injections which gave significant relief in its symptoms [34].

A case study by Bârzu et al. reported different diagnostic tests for identifying piriformis syndrome especially for those patients who cannot afford magnetic resonance imaging, computerized tomography, etc. One of the main parts of assessment is observing the pattern of gait and posture and then performing tests like the FAIR test, Beatty maneuver, modified Beatty maneuver, Freiberg test, Pace test, modified Pace test, straight leg raise, Mirkin test, Bragard test, Bonnet test, and piriformis muscle stretching [35].

As per a study conducted by Laha et al. on 30 patients who were randomly divided into two groups, one group received strengthening exercises for hip extensors and abductors, piriformis release, and nerve mobilization, while the other group was given only stretching and nerve mobilization. Pain was graded on the NPRS and functional status for lower extremity was measured. They concluded that adding hip strengthening had a higher beneficial effect than just stretching and mobilization [36].

The summary of the above discussion of different articles is presented in Table 1.

**Table 1:** Summary of discussion FAIR: Flexion abduction internal rotation; US: ultrasound; PIR: post-isometric relaxation; RI: reciprocal inhibition; MRI: magnetic resonance imaging; CT: computerized tomography

Author/year	Design	Sample	Aim	Intervention	Conclusion
Kirschner et al., [30] 2009	Review	One subject	To study the diagnostic measures and treatment protocol	Myofascial release, stretching, anesthetics, and corticosteroid injections	Physiotherapy maneuvers have proven to be effective in treating piriformis syndrome.
Michel et al., [31] 2013	Experimental design	Two-hundred and fifty patients	To develop treatment strategies and diagnose piriformis syndrome	Deep transverse massage, pelvic-trochanter muscle stretch, self-rehabilitation techniques, and proprioceptive pelvic femoral exercises.	Deep transverse massage, pelvic-trochanter muscle stretch, self-rehabilitation techniques with proprioceptive pelvic femoral exercises and stretching with Onabotulinumtoxin A injections showed decrease in symptoms
Fusco et al., [32] 2018	Case series	Three patients with complaints of piriformis syndrome	Effect of ultrasound (US) guided dry needling to release piriformis muscle	US-guided dry needling	A definite decrease in symptoms of piriformis syndrome
Bose et al., [33] 2018	Comparative study	Forty-five subjects	Correlate the effects of post-isometric relaxation (PIR) and reciprocal inhibition (RI)	PIR and RI	Increased range of motion at hip joint, decreased pain, and better functional outcome were found with PIR and conventional physiotherapy
Norbury et al., [34] 2012	Case report	One patient	Discuss the approach of a clinician to evaluation and treatment of this patient.	Relaxing tight muscles, activity modification, lidocaine, and botulinum toxin A injections	It showed that there was a definite reduction in pain with botulinum toxin A injection and stretching with activity modification.
Bârzu et al., [35] 2013	Case study	One sample	To study the diagnostic method in piriformis syndrome	FAIR test, Beatty maneuver, Modified Beatty maneuver, Pace test, Freiberg test, modified pace test, straight leg raise, Bragard test, Bonnet test, Mirkin test, and piriformis muscle stretching	Even without the availability of MRI, CT, etc. piriformis syndrome can be diagnosed with simple functional tests. These tests reproduce pain by stretching or over contracting the muscle compressing the nerve.
Laha et al., [36] 2018	Randomized clinical Trial	Thirty subjects with piriformis syndrome	To find out the improvement for pain intensity and functional status in group A and group B	Hip abductor strengthening, hip extensor strengthening, sciatic nerve mobilization, and piriformis stretching	Both the groups showed a significant decrease in pain and improved the lower extremity function by giving hip extensor and abductor strengthening combined with piriformis stretching and neural mobilization.

## Conclusions

After studying various articles about the different approaches for the management of piriformis syndrome, stretching on a regular basis has been shown to be quite effective for this condition and for improving the functional activity of hip articulation. Neural mobilization has been found to be one of the primary interventions for symptomatic relief. It reduces the irritation of the sciatic nerve occurring due to the spasm of piriformis muscle. Although this technique provides a transitory relief, when combined with muscle stretch, it gives a better and faster outcome. Hence, it can be said that piriformis release and sciatic nerve mobilization both are effective individually.
